# The Intersection of SARS-CoV-2 and Diabetes

**DOI:** 10.3390/microorganisms13061390

**Published:** 2025-06-14

**Authors:** Jacob H. Nichols, Amber M. Smith, Colleen B. Jonsson

**Affiliations:** 1 Department of Microbiology, Immunology, and Biochemistry, University of Tennessee Health Science Center, Memphis, TN 38163, USA; jnicho60@uthsc.edu (J.H.N.); amber.smith@uthsc.edu (A.M.S.); 2 Department of Pediatrics, University of Tennessee Health Science Center, Memphis, TN 38163, USA

**Keywords:** SARS-CoV-2, diabetes, disease modeling, pathogenesis, in vitro models

## Abstract

The interplay between comorbidities and viral infections is a critical factor that influences disease severity and outcomes. Diabetes Mellitus (DM) is one such comorbidity that significantly elevates the risk of severe viral infection from coronaviruses, namely, SARS-CoV-2. DM is characterized by either a lack of insulin production (type 1 diabetes) or insulin resistance (type 2 diabetes), both of which contribute to a state of hyperglycemia, or high blood sugar. Hyperglycemia significantly promotes chronic inflammation, metabolic dysfunction, and immune dysregulation, which put diabetics at an elevated risk of critical health outcomes. Additionally, diabetes is hypothesized to amplify viral titers during infection by promoting the expression of the viral entry receptor ACE2 and providing a favorable cellular energy environment for viral replication. This review focuses on explaining the mechanisms that link diabetics with more severe COVID-19 disease and exploring some of the mechanisms that contribute to the phenomenon where COVID-19 can promote new-onset diabetes. By highlighting the interconnections between diabetes and COVID-19, this review aims to emphasize the implications that the SARS-CoV-2 outbreak has had on metabolic health.

## 1. COVID-19

In late 2019, severe acute respiratory syndrome coronavirus 2 (SARS-CoV-2) spilled over into the human population from an unknown zoonotic source in Wuhan, China, and resulted in a global pandemic of coronavirus disease-2019 (COVID-19) [1,2,3]. Since then, more than 1.2 million people have died from COVID-19 in the United States alone (CDC COVID data tracker). SARS-CoV-2 is a positive-sense, single-stranded RNA virus that belongs to the genus *Betacoronavirus* (β-CoV). β-CoVs have garnered significant public interest that began with SARS-CoV (SARS) and then MERS-CoV (MERS) spilling over into human populations from zoonotic reservoirs and causing outbreaks [4]. The SARS-CoV epidemic caused an outbreak that began in the Guangdong province of China and spread to nearly 30 other countries in 2002–2003 [5]. Similarly, the MERS-CoV outbreaks occurred from 2012 to 2015 in the Middle East, and there was also a large outbreak in South Korea in 2015 that originated from a single person who had traveled to the Middle East [6,7]. Like SARS-CoV, the primary receptor for SARS-CoV-2 is the angiotensin-converting enzyme-2 (ACE2) receptor [8,9]. ACE2 is a highly expressed protein receptor in the upper and lower respiratory epithelium but is also expressed in other tissues, including the kidneys and intestines [10]. Although SARS-CoV-2 infections in humans primarily take place in the upper and lower respiratory tracts, there is strong evidence that infection occurs in the liver [11], intestinal tract [12], brain [13], and pancreas [14]. SARS-CoV-2 infections can cause oxygen and metabolic changes that contribute to damage to the heart, brain, kidney, and vascular tissues [15,16].

COVID-19 symptoms range from asymptomatic to severe [17,18]. Mild symptoms include fever, chills, body aches, and loss of smell, while severe symptoms include shortness of breath, chest pain, and intense coughing that can progress to acute respiratory distress syndrome (ARDS). If symptoms worsen, patients may require supplemental oxygen or intratracheal ventilation. Vascular dysfunction, such as blood clots and thrombosis, can also manifest as a result of COVID-19 [19]. The long-lasting impacts of COVID-19, termed long-COVID, are well-documented and may present as neurological and cardiovascular sequelae [20,21,22]. Acute and long-term COVID-19 complications can be especially devastating to individuals with pre-existing conditions that put them at a health disadvantage. This review will focus on the intersection of COVID-19 with diabetes, a very common comorbidity. Table 1 highlights some of the studies on COVID-19 patients discussed herein with respect to diabetes.

## 2. Diabetes Has Emerged as an Important Comorbidity That Markedly Increases the Severity of COVID-19

Diabetes incidence is on the rise worldwide, affecting both affluent and poor communities in emerging and developed nations [39,40]. According to the CDC National Center for Health Statistics, it was the seventh leading cause of death among Americans in 2023 [41], and almost 16% of the adult population was living with diabetes as of 2023 [42]. Diabetics cannot regulate their blood glucose levels because of insufficient insulin production (type 1 diabetes) or insulin insensitivity (type 2 diabetes). Hyperglycemia, or high blood glucose, is the main factor from which the downstream complications of diabetes stem [43,44]. Specifically, hyperglycemia primarily affects vascular tissue and can lead to sustained inflammation, organ failure, and nephropathy [43,44,45]. This constellation of disease manifestations that contribute to diabetes are recognized as key factors that significantly increase the risk of severe COVID-19 [23,24]. As such, diabetes is considered an immunocompromising disease [46].

### 2.1. Meta-Analysis of Diabetes Representation Among Persons with β-CoVs (Epidemiology)

Diabetes emerged as a prominent comorbidity associated with SARS and MERS [47,48]. During the first SARS epidemic in 2002–2003, diabetes was associated with mortality in Hong Kong [48]. In a study of hospitalized patients in Beijing during the 2003 SARS epidemic, the percentage of patients with a diabetes diagnosis was higher in the deceased group than the survivor group, indicating an association between diabetes and risk of death [49]. Additionally, infection with SARS-CoV caused an increase in fasting plasma glucose compared to uninfected control patients [50]. Data from the MERS epidemic shows that diabetes status was significantly associated with contracting MERS illness [51]. Moreover, diabetes was significantly associated with a higher mortality rate in MERS-CoV-infected individuals [52].

Similar conclusions have been drawn with respect to increased COVID-19 hospitalization rates for individuals with diabetes. In a cohort of 5700 COVID-19 patients from the metropolitan area of New York City, diabetes stood as the third most common comorbidity at roughly 33%, which was behind hypertension (57%) and obesity (42%) [25]. In a study in England, the risks of comorbidities in COVID-19 cases were assessed by surveying the health records of 61,440,470 individuals [26]. Analyses of the COVID-19-related deaths that occurred in-hospital between 1 March and 11 May of 2020 found that 31.4% of individuals had type 2 diabetes and 1.5% had type 1 diabetes. This is in contrast to the proportions in the entire sample group, where they found that 4.7% had type 2 diabetes and 0.4% had type 1 diabetes. These findings indicate that both type 1 and type 2 diabetes were associated with an increased risk of mortality from COVID-19.

There have been mixed findings on the protection afforded by previous SARS-CoV-2 infection or vaccination in diabetic patients. In a meta-analysis of the outcomes of 17 different published articles, vaccine efficacy had significantly waned in the diabetic population compared to healthy controls [27]. Unsurprisingly, vaccinated diabetics had a significantly higher odds ratio for SARS-CoV-2 breakthrough infection compared to healthy controls. This was supported in animal studies, where humoral responses induced by the BNT162b2 mRNA vaccine were reduced in an HFD diabetic model of C57BL/6J mice compared to healthy controls [53]. However, other studies in humans found that the humoral response in a vaccinated diabetic cohort was similar to that of a vaccinated non-diabetic control cohort [28].

### 2.2. Why Diabetes Contributes to Worse Outcomes

Hyperglycemia induces the generation of reactive oxidative stress (ROS) factors, which in turn promote inflammation [54]. Additionally, diabetes is often accompanied by obesity, which likewise promotes a high basal level of inflammation. Visceral adipose tissue promotes a significantly pro-inflammatory environment in the individual and leads to secretion of TNF-α, IL-6, IL-8, and adipokines that may amplify other immune responses [55,56,57]. This can be particularly deadly, especially for SARS-CoV-2, if it leads to a “cytokine storm”, which is an outsized, dysregulated immune response to the virus. The excess or overproduction of these immune factors can cause acute respiratory stress and organ damage in severe COVID-19. The blockade of IL-6 in particular has shown some success as a treatment option for severe and critical COVID-19 in clinical trials [58].

Diabetic panvascular disease (DPD) is another disorder that has a severe impact on the normal homeostatic function of multiple organs, including the heart, kidneys, and lungs [59]. DPD manifests as macro- and microvascular atherosclerosis, which contributes to endothelial damage in the aforementioned organs [60]. Hyperglycemia has been implicated as a leading factor in inducing lung injury in diabetics [61]. Preexisting injury in the lungs and other organs puts diabetic individuals at a disadvantage when fighting a viral infection.

### 2.3. Clinical Findings and Indicators Associated with Worse Outcomes (Pathogenesis)

The immunocompromising factors (i.e., vascular damage in the lung, and pro-inflammatory microenvironment) of diabetes are proposed to increase the risk of death and severe pathology from COVID-19 [29]. A study in a Swedish cohort found that type 2, but not type 1, diabetes was independently associated with an elevated risk of hospitalization for COVID-19 patients [30]. Elevated HbA1C levels in the type 2 diabetic cohort were associated with a higher risk of worse outcomes. In addition, a higher body mass index (BMI) index was independently associated with a higher risk of worsened outcomes. This would imply that uncontrolled diabetes and diabetes-caused obesity promote worsened disease.

A retrospective study in the USA analyzed the risk of death based on HbA1c levels among T2D COVID-19 patients and found that the risk of death increased with increasing HbA1c levels [31]. A Japanese study of SARS-CoV-2-infected individuals in 2020–2021 reported that the proportion of critical outcomes (supplemental oxygen, invasive ventilation, or death) was higher in the diagnosed and undiagnosed diabetic cohorts, 30% and 35%, respectively, compared to the non-diabetic group, where it was 5% [32]. Additionally, the proportion of undiagnosed and diagnosed diabetics who accumulated complications, such as bacterial infections, thromboembolism, or acute kidney injury, was also higher than recorded in the non-diabetic group. High HbA1c levels, a measurement of average blood glucose concentration, correlated to worse COVID-19 outcomes. Lastly, a study in Italy found a higher abundance of SARS-CoV-2-positive staining in postmortem cardiac tissue of diabetics [33].

## 3. The Incidence of Diabetes Is Higher in Individuals After COVID-19

SARS-CoV, the causative agent of the 2002 epidemic, was correlated with elevated plasma glucose levels. Two studies examined cohorts of individuals hospitalized with SARS-CoV and found that their fasting plasma glucose levels were elevated compared to the non-SARS-CoV-infected groups [49,50].

Similarly, COVID-19 has been reported to induce hyperglycemia and new-onset diabetes, where a systematic review of new-onset diabetes after COVID-19 in the USA, Germany, and England found a significant association [34]. SARS-CoV-2 infection was associated with a rise in the incidence of diabetes, hyperlipidemia, and hypertension [34]. Vaccinated cohorts did not have a rise in diabetes, hyperlipidemia, and hypertension [35]. A 551-patient cohort of COVID-19 in Italy additionally found a significant correlation between the patients and their hyperglycemic status. Patients who had recorded hyperglycemia upon hospital admission had persistent hyperglycemia for at least 6 months [36]. They reported individuals with no history of diabetes presenting with new-onset diabetes and insulin resistance after having COVID-19. A study of over 700 patients from Brazil who had been hospitalized with COVID-19 from March to August 2020 found that the proportion of hyperglycemia incidence was higher in the COVID-19-positive patients than the COVID-19-negative patients, regardless of diabetes diagnosis [11]. Not only does this phenomenon occur in adults, but it has been observed in pediatric populations as well, where a cohort of over 600,000 patients aged 10–19 revealed that the risk of new-onset diabetes was raised in pediatric patients within 6 months of COVID-19 diagnosis [37].

### 3.1. Potential Mechanisms Proposed for New-Onset Diabetes Post-COVID-19

SARS-CoV-2, like all viruses, relies on host cell machinery to replicate its genetic material and complete its life cycle. A hallmark of viruses is their ability to hijack cellular functions to promote their viral life cycle. A common target of viruses is the host’s central carbon metabolism pathways.

Central carbon metabolism refers to the interconnected biochemical cycles that produce energy from carbohydrates. Mammalian cells primarily rely on aerobic respiration to metabolize glucose into usable energy, ATP. In brief, glucose is shuttled into the cell and immediately enters the cycle of glycolysis and generates two new molecules of ATP and one molecule of acetyl-CoA, the input for the citric acid cycle. The process occurs in the cytosol independent of oxygen and can compensate for energy production in the cell when oxygen is present at sub-optimally low concentrations. Aerobic respiration is the most efficient method of glucose metabolism in the presence of oxygen. The citric acid cycle produces succinate, which undergoes oxidative phosphorylation (OXPHOS) in the cristae of the mitochondria and generates the bulk product of ATP (36 molecules). It is important to note that additional byproducts are made throughout these metabolic processes, including serine, glycine, and nucleotide precursors. The pentose phosphate pathway runs in tandem with glycolysis and is responsible for the production of ribose-5-phosphate, an important precursor to DNA and RNA.

It should come as no surprise that viruses commonly target host bioenergetic systems to promote their replication. The exact mechanism of how viruses hijack these systems varies, but one hypothesis proposes viruses modulate host pathways to increase the production of nucleotides and other substrates/macromolecules that are necessary for the assembly of mature virions [62]. For example, influenza A virus (IAV) infection induces glycolysis in vitro and in vivo [63,64]. An association between glycolysis activity and IAV titers was demonstrated, where inhibition of glycolysis prevented viral replication [64]. There are many more examples of viruses that increase glycolysis, including Zika virus [65], dengue virus [66], and human cytomegalovirus [67].

Another important group is the oncogenic viruses, such as human papillomavirus (HPV), hepatitis B virus (HBV), and Epstein–Barr virus (EBV), which activate host cell glycolysis in what is known as the ‘Warburg effect’ [68]. The Warburg effect is the phenomenon in which cancer cells have a heightened dependency on energy generation by glycolysis, unlike non-cancerous cells, which depend primarily on aerobic respiration and OXPHOS. Oncogenic viruses contribute to a cell becoming cancerous by activating oncogenes, but they simultaneously activate host cell glycolysis, mirroring the Warburg effect that can be observed in cancer cells [69]. The propensity for viruses to interfere with host cell metabolism could explain the new onset of diabetes after COVID-19.

### 3.2. SARS-CoV-2 Targets Mitochondrial Function

In A549 cells, a continuous lung adenocarcinoma cell line, transfection of plasmid expressing the nonstructural proteins (nsp) of SARS-CoV-2, *ORF9b*, *ORF9c*, and *ORF10* interact directly with mitochondrial proteins [70]. The nsp produced by *ORF3a* downregulated the expression of proteins that have a role in maintaining mitochondrial function [70]. Expression of the nsp from *ORF9b* promoted fragmentation of mitochondria, and expression of the nsp from the ORF9c also altered cristae structure. The nsp from the expression of *ORF10* promoted the fusion of mitochondria. The study was limited to only being shown in vitro, but it does provide a starting point to further investigate the role of the viral proteins in the alteration of host mitochondrial function.

Comparing RNA-Seq data across SARS-CoV-2-infected A549, A549-ACE2, Calu-3, and NHBE cells in addition to lung tissue from autopsies and bronchoalveolar lavage fluid (BALF) from patients suggested that SARS-CoV-2 downregulated mitochondrial ribosome protein genes in primary cell culture compared to IAV [71]. Mitochondrial Complex 1 gene and cellular respiration-related genes were downregulated in SARS-CoV-2-infected cell lines compared to cells infected with IAV, human parainfluenza viruses (HPIV), or respiratory syncytial virus (RSV). In nasopharyngeal samples from infected patients, mitochondrial OXPHOS gene expression was downregulated and correlated with the peak of viral RNA levels [72]. An inverse association was also observed with OXPHOS gene expression recovering as the viral RNA in the sample declined.

### 3.3. Disruption of Glucometabolic Control and Promotion of Glycolysis After SARS-CoV-2 Infection

**Figure 1:**
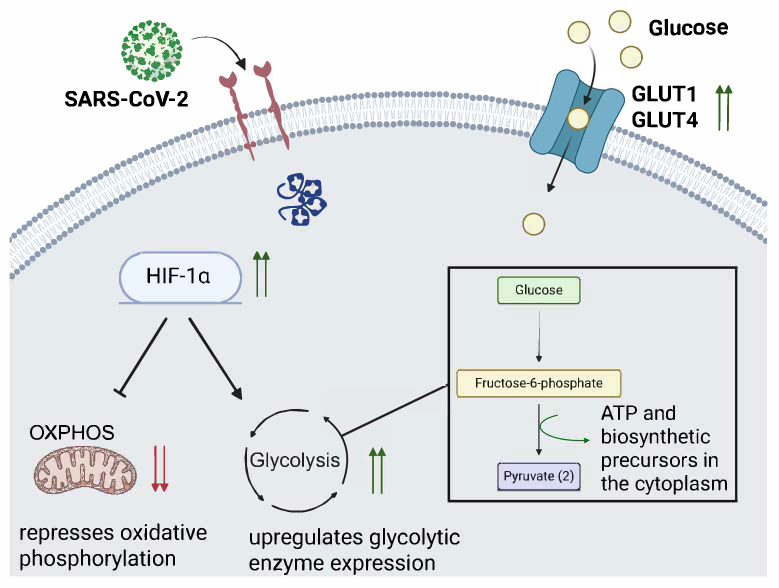
SARS-CoV-2 infection promotes HIF-1α activity and glucose transporter expression. The illustration depicts a proposed model by which SARS-CoV-2 infection promotes glycolysis through upregulation of hypoxia inducible factor 1 alpha (HIF-1α) and glucose transporters (*GLUT1* and *GLUT4*). Green upwards arrows indicate HIF-1α expression is increased, which drives glycolysis, shown by the black arrow. The bar-headed arrow emerging from HIF-1α indicates that oxidative phosphorylation activity is decreased by HIF-1α activity. *GLUT1* and *GLUT4* are pictured on the surface of the cell with two green arrows also indicating their increased expression from SARS-CoV-2 infection. The two red arrows indicate decreased expression.

SARS-CoV-2 infection has been shown to affect the protein expression of two major glucose importers, *GLUT1* and *GLUT4*, (Figure 1) in the lung and cardiac tissue in a feline model [73]. The group infected with the SARS-CoV-2 Delta variant had increased *GLUT1* expression in the heart and lung tissue at 4 days post-infection (dpi) and 12 dpi, and the *GLUT4* insulin-sensitive glucose transporter was increased in heart and lung tissue at 4 dpi [73]. In infected Vero E6 TMPRSS2 cells, glucose and folate were depleted after SARS-CoV-2 infection, while lactate was increased, suggesting that glycolysis was increased [74]. Conversely, galactose supplementation did not promote SARS-CoV-2 replication. It is important to note that galactose is an input for the OXPHOS cycle, while glucose can be used for glycolysis.

During SARS-CoV-2 infection, the accessory protein *ORF3a* was shown to promote Hypoxia-inducible factor-1 (HIF1α) production in a Caco2 cell culture [75]. Additionally, HIF-1α expression was higher in peripheral blood mononuclear cells (PBMCs) from COVID-19 patients than in healthy patients. HIF-1α boosts glycolysis activity and can be leveraged by other viruses, such as RSV, to promote their replication [76].

It has also been reported that SARS-CoV-2 infection upregulates the HIF1α-dependent glycolysis axis in monocytes [77]. Monocyte dysfunction has been identified as a major driver of acute COVID-19 [78,79]. Inhibitors of *PFKFB3* (3-PO), glycolysis (2-DG), and LDH-A (Oxamate) block SARS-CoV-2 replication in monocytes. However, an inhibitor of the mitochondrial pyruvate carrier was unable to do the same, implying that TCA cycle activity is not important for viral replication. IAV and RSV increased glycolytic activity in monocytes to a lesser extent than SARS-CoV-2 [77]. This would, in effect, induce aerobic glycolysis to sustain viral replication in human monocytes.

Using a model of SARS-CoV-2 infection in human pluripotent stem cell (hPSC)-derived organoids, which were differentiated into airway organoids, an inhibitor of glycolysis (GW6471) was able to block SARS-CoV-2 WA1 infection [80]. Chetomin and shRNA specific for HIF1 α also blocked SARS-CoV-2 infection. Metabolic profiling showed upregulation of D-glucose-6 phosphate, citric acid, fatty acids, and amino acids, suggesting that SARS-CoV-2 infection promoted glycolysis in the hPSC model. This phenomenon has been observed in other viral infections, including RSV upregulation of HIF1α [76].

SARS-CoV-2 could induce a shift from the OXPHOS cycle to glycolysis to support its proliferation. In doing so, the host cell’s glucometabolic control would be significantly skewed towards glycolysis, and as more cells and tissues are infected, so too would the glucometabolic dysregulation. The hypothesis is that widespread glycolysis upregulation would require a high amount of insulin to promote glucose uptake by the infected cells. Sustained high levels of insulin could promote insulin resistance in the host and contribute to new-onset type 2 diabetes diagnosis even after recovery.

### 3.4. COVID-19 Can Cause Direct and Indirect Damage to the Pancreas

Pancreatic dysfunction is often a key contributor to type 1 diabetes. The pancreas produces the insulin necessary to regulate the body’s blood glucose levels. However, in cases of severe autoimmunity or insensitivity from an unhealthy diet, it can fail to maintain acceptable blood glucose levels and lead to an individual’s diabetes diagnosis.

In a cohort of infected and infected + vaccinated non-human primates (NHP), the elder NHPs had a higher immunopositivity for the spike protein after infection compared to the adult NHPs, in addition to more severe lesions and amyloid deposits in the pancreas, hallmarks of type 2 diabetes cases [81]. Another group conducted a histological analysis of pancreas sections from COVID-19 autopsy patients and found more extensive microthrombi, fibrosis, and endothelialitis compared to the samples from the uninfected patients [14].

By infecting pancreatic islets collected from healthy donors with SARS-CoV-2, it was shown that they are susceptible to infection and subsequent β cell impairment and death [82]. Histological samples from COVID-19 patient autopsy tissue also found that the pancreatic beta cells stained positive for SARS-CoV-2 nucleoprotein (NP) in 7/9 patients confirmed COVID-19 positive by PCR. Another study conducted immunostaining on tissues from COVID-19 patient autopsy tissue and found SARS-CoV-2 infection in pancreatic β cells as well [14]. Infection of these cells would likely be accompanied by immune cell infiltration. There was one reported case of new-onset type 1 diabetes after COVID-19 in a 19-year-old patient who had severe β cell dysfunction for weeks after the infection cleared [83]. The authors concluded that the diabetes diagnosis was causally linked to the direct damage of the pancreas β cells as a result of the SARS-CoV-2 infection.

Other viruses have been speculated to cause new-onset type 1 diabetes in patients by directly damaging the pancreas [84], but larger cohort studies are needed to fully characterize the effect. Likewise, more studies are necessary to determine the extent to which SARS-CoV-2 can cause sufficient pancreatic damage to promote diabetes.

### 3.5. COVID-19 Vaccination Has Been Speculated to Induce Latent Autoimmune Diabetes in Adults (LADA)

LADA is a slow-onset version of diabetes that shares some characteristics with both type 1 and type 2 diabetes. Similar to type 2 diabetes, LADA is typically acquired in individuals over the age of 30, and as such, patients are often initially misdiagnosed as having type 2 diabetes. Unlike type 2 diabetes and similar to type 1 diabetes, it is immune-mediated, where autoimmune damage of an individual’s pancreatic β cells caused by a combination of CD8^+^ T cells, CD4^+^ T cells, B cells, macrophages, and dendritic cells impairs pancreas function [85]. Importantly, COVID-19 vaccination with the mRNA vaccine BNT162b2 can give rise to other autoimmune diseases, such as Graves disease [86]. It has also been speculated to drive the development of LADA in one documented case where a prediabetic individual who, soon after receiving the second dose of BNT162b2, had been admitted to a hospital for a diabetic ketoacidosis episode and tested positive for autoantibodies [38]. There would need to be larger-scale studies to establish a clear causal relationship between COVID-19 vaccination or infection and the induction of LADA, but it should be considered as a potential contributing factor to new-onset diabetes.

## 4. In Vivo Models Used to Study SARS-CoV-2 Infection in a Diabetic Host

Three main models of diabetes-induced mice have been used to study SARS-CoV-2 infection. They include the high-fat diet mice (HFD), db/db mice, and streptozotocin (STZ)-induced diabetic mice. Each approach simulates diabetic conditions, but they differ slightly in the pathogenic outcomes. Additionally, special attention should be given to whether the study used a mouse-adapted strain of SARS-CoV-2 or if it was propagated from a human isolate, as this can impact interpretations.

### 4.1. Db/Db (Leptin Deficient) Mouse Model

Db/db mice are deficient in the leptin receptor [87]. These mice are less capable of lowering blood glucose levels and have dysregulated metabolism. The C57BL/6J db/db animal model presents local swelling of the liver and worse pathological scores in the lung, liver, and pancreas compared to the uninfected and infected WT mice, indicating that the db/db model recapitulates severe diabetic complications [88]. Infecting C57BL/6J db/db mice with SARS-CoV-2 B.1.1.7 (Alpha Variant of Concern) showed that they were more susceptible to infection and severe pathological damage as compared to the wild-type (WT) mice [88]. The infected C57BL/6J db/db mice had worse pathological scores based on H&E staining of lung tissue, which included more intense pulmonary hemorrhage. Additionally, the SARS-CoV-2 Alpha-infected C57BL/6J db/db mice had higher viral gRNA recovered from lungs and throat swabs compared to the WT group.

The K18-hACE2 db/db mouse model, which is transgenic for the human ACE2 receptor, also suggested that infection with SARS-CoV-2 is enhanced in a diabetic host. Infecting these animals with the SARS-CoV-2 strain hCoV-19/China/CAS-B001/2020 (CAS-B001) showed that blood glucose levels were slightly higher in the diabetic group compared to the non-diabetic group [89]. Staining for SARS-CoV-2 N protein in the lung tissue showed that the diabetic mice supported more widespread virus replication with higher histopathological scores. Cathepsin L (CTSL) activity in db/db mice suggested that CTSL, a cofactor that promotes SARS-CoV-2 entry, is more active in the db/db mice [90]. This could explain why virus replication was elevated in these animals.

### 4.2. HFD Mouse Model

The HFD mouse model offers a more natural model of diabetes that has been caused by lifestyle. The mice are restricted to eating chow with a higher fat content and experience significant weight gain and become glucose-resistant after about three months on the diet. When diabetes was induced in C57BL/6 mice by keeping them on an HFD for 12–17 weeks, infecting them with MERS-CoV resulted in prolonged histopathological inflammation in the lung tissue compared to non-diabetic mice but had similar levels of virus [47]. Immunizing HFD animals with the mRNA vaccine BNT162b2 or a recombinant nanoparticle vaccine (NVX-CoV2373) and then infecting them at least two weeks later with a mouse-adapted strain of SARS-CoV-2 (MA/10) suggested virus clearance was delayed in the HFD group compared to the CD group. In the non-vaccinated animals, there was increased mortality, but not viral loads, in the HFD group compared to the control diet (CD) group after infection [53,91].

In contrast, K18-hACE2 mice with HFD-induced diabetes exhibited a similar course of infection to the CD group based on viral quantitation by qPCR at 3 dpi and histopathological analysis after infection with SARS-CoV-2 (hCoV-19/China/CAS-B001/2020) [89]. This implies that important differences between the model implementations, such as mouse and virus strain, can lead to data interpretations that vary widely.

### 4.3. Streptozotocin (STZ) Mouse Model

STZ induces diabetes in mice by damaging the pancreatic beta cells of the mouse and thereby reducing insulin production. Because the mouse cannot generate insulin to lower blood glucose levels after STZ treatment, it is considered a model of type 1 diabetes. There are limited studies that have been published on the effect of STZ injection on SARS-CoV-2 infection. However, one study used a model where hACE2 knock-in mice were injected with STZ, and then the animals were switched to an HFD for eight weeks to induce diabetes. Intravenously injecting the SARS-CoV-2 spike and nucleoprotein into the jugular significantly increased TLR signaling (TLR8, MyD88, and TRAF6) in the diabetic mice compared to control mice [92].

A study using STZ-treated mice as a diabetic model to study their reaction to S-protein immunization showed that STZ mice had a reduced IgG reaction at 7 weeks post-immunization compared to the non-diabetic mice [93]. However, mice were not subsequently challenged with the virus. Similar to what was found in db/db mice, induced diabetes in BALB/c mice using STZ and treating with insulin showed that CTSL, the host protease that exposed the Spike fusion peptide, mRNA expression was increased in diabetic mice [94].

## 5. In Vitro Models of SARS-CoV-2 Infection in Diabetic Conditions

While in vivo animal models of diabetes are useful for recapitulating human disease, in vitro models are important to highlight the cellular mechanisms at play. There are, however, fewer reports that use in vitro cell culture models of diabetes to study SARS-CoV-2 infection.

### 5.1. Continuous Cell Lines

It has been reported that ACE2 mRNA expression increases with increases in glucose concentration in a continuous lung epithelial cell line, Calu-3 cells [95]. BAY-876, a *GLUT1* inhibitor, could prevent the increase in ACE2 mRNA expression, indicating that glucose influx into the cell leads to ACE2 mRNA upregulation. Similarly, one study showed that high glucose promoted mRNA expression of ACE2 in A549 cells [96], while another showed that D-galactose significantly increased the expression of cell receptors FURIN, TMPRSS2, and ACE2 [97].

Since ACE2 is a receptor for SARS-CoV-2 entry, these data suggested that persons with hyperglycemia may show greater susceptibility to SARS-CoV-2 infection. The increased expression of all three receptors is highly expected to increase susceptibility to SARS-CoV-2 infection. Indeed, there are in vitro studies that report that higher levels of glucose result in higher levels of virus production. In a study that infected monocytes with an isolate of SARS-CoV-2 from the second confirmed case in Brazil (GenBank: MT126808.1) and then supplemented the cells with increasing glucose concentrations (0, 2.5, 5.5, 11.1, and 22.2 mM), viral loads increased in association with glucose concentration after 24 h [77]. The increased viral load was attributed to the HIF-1α regulation of glycolysis.

High glucose also promoted infection with a SARS-CoV-2 pseudovirus in immortal human liver cells, Huh7, but not in CTSL knockout cells [90]. Greater pseudovirus infection in Huh7 cells grown in sera from diabetic donors has been observed, with high glucose correlating with higher CTSL activity, which may promote SARS-CoV-2 infection.

### 5.2. Primary Kidney and Brain Cell Lines

Using cells isolated from human donors to study SARS-CoV-2 infection increases the translatability of findings from other in vitro and in vivo models. In one study, human proximal tubular cells (HPTCs) isolated from the kidneys of diabetic individuals had higher basal respiration, ATP production, and ACE2 expression [98]. The cells also stained more positively for SARS-CoV-2 infection in addition to higher SARS-CoV-2 mRNA. Treating the cell samples with dichloroacetate (DCA), an inhibitor of pyruvate dehydrogenase kinase (PDK), decreased SARS-CoV-2 mRNA expression, suggesting that glycolysis activity is important for infection.

Culturing of human brain microvascular endothelial cells in either normal or high glucose medium and treating with SARS-CoV-2 spike protein suggested that cells grown in high glucose had higher concentrations of IL-6, TNF-α, and IL-1B compared to cells grown in a normal glucose environment [92].

### 5.3. Kidney Organoid

Organoids are growing in popularity due to greater sophistication as compared to 2D cell culture models and less intensive handling procedures compared to animal models. One group modeled SARS-CoV-2 infection in a kidney organoid model, which was subjected to high glucose oscillations (25mM and 5mM oscillated) to induce a diabetic state [98]. In their model, high glucose oscillations promoted the buildup of fibronectin and collagen fiber deposits, which mirrors some of the pathology consistent with chronic kidney disease and renal fibrosis, which can affect diabetics [99,100]. Hexokinase 2 (*HK2*) and *LDHA* genes, important enzymes in glycolysis, were upregulated [98]. Additionally, high glucose oscillation promoted gene and protein expression of ACE2. They found that SARS-CoV-2 infection was promoted by the high glucose oscillations compared to the low glucose control group at 1 dpi.

## 6. Discussion

Along with the elderly and immunocompromised populations, diabetics are considered at high risk of developing severe COVID-19. In general, clinical trials, animal studies, and cell-based models suggest that there is a strong bidirectional relationship between COVID-19 and diabetes. Clinical studies indicate that individuals with diabetes face more severe symptoms of COVID-19, with diabetes being one of the most overrepresented comorbidities among individuals hospitalized with COVID-19. SARS-CoV-2 has also been shown to replicate to higher titers in diabetic animal models and to associate with glucose concentration in cell culture models. Several animal models have revealed that vaccinated diabetic mice take longer to clear the virus after infection compared to control mice. Additionally, cell culture models have been used to demonstrate that hyperglycemia could promote SARS-CoV-2 infection and replication in the host cell. Additionally, there is a heightened proinflammatory response in diabetic mice.

Animal and cell culture models have provided ample evidence that SARS-CoV-2 infection dysregulates the metabolic cycle in the host at the cellular level and across tissues. This offers a strong foundation for the hypothesis that implicates COVID-19 as a contributor to new-onset diabetes in recovered patients. Special attention should continue to be placed on seeking out more efficient therapeutics, especially for the diabetic population, to counteract their overrepresentation in hospitalized cohorts. It should also be emphasized by medical professionals that those who overcome severe COVID-19 should monitor their blood glucose for a period after recovery to mitigate the incidence of new-onset diabetes. Additionally, there is an obvious gap in sophisticated in vitro models that can replicate the intracellular and intercellular hallmarks of diabetes, their susceptibility to SARS-CoV-2 infection, and the host–pathogen interactions of SARS-CoV-2 infection and diabetes.

## Figures and Tables

**Table 1:** Clinical studies of COVID-19 patients and diabetes.

Year	Country	Conclusions	Reference
2020	USA	COVID-19 patients with diabetes had significantly greater levels of inflammatory biomarkers and risk of severe pneumonia than COVID-19 patients who were not diabetic.	[23]
2020	China, Italy, USA	Meta-analyses of clinical cohort studies indicate that diabetes is associated with a greater incidence of COVID-19. Additionally, severity is increased in diabetic COVID-19 patients compared to non-diabetic patients.	[24]
2020	USA	In a cohort of 5700 hospitalized COVID-19 patients, diabetes was the third most common comorbidity (33.8%), behind obesity (41.7%) and hypertension (56.6%).	[25]
2020	England	Type 1 and type 2 diabetes independently increased a COVID-19 patient’s odds of in-hospital death.	[26]
2022	International	Meta-analysis of 17 clinical studies conducted across the world revealed that SARS-CoV-2 vaccine effectiveness was diminished in diabetic cohorts compared to non-diabetics.	[27]
2022	Austria and Germany	Type 1 and type 2 diabetics who had been vaccinated had a similar humoral immune response to the SARS-CoV-2 receptor binding domain (RBD) compared to healthy individuals.	[28]
2020	International	A group in Italy conducted a meta-analysis which revealed that severe outcomes such as thromboembolism and reduced lung function were predominantly seen in type 2 diabetic COVID-19 patients.	[29]
2021	Sweden	There was observed a higher risk of severe COVID-19 outcomes associated with raised glycated hemoglobin (HbA1c) levels. Type 2 diabetes was associated with a greater risk of hospitalization, intensive care, and death.	[30]
2022	USA	The risk of hospitalization, invasive ventilation, and death from COVID-19 is increased with increasing HbA1c levels.	[31]
2022	Japan	Undiagnosed diabetes, prediabetes, and diagnosed diabetes were significant risk factors for severe COVID-19 outcomes. Additionally, increased HbA1c levels were associated with COVID-19 severity.	[32]
2021	Italy	Myocardial tissue from autopsy tissue of patients who died from COVID-19 had a higher percentage of positive staining for SARS-CoV-2.	[33]
2022	USA, England, Germany	Meta-analysis of patients after recovering from COVID-19 revealed an increased incidence and risk of developing new-onset diabetes.	[34]
2023	USA	Patients who had recovered from COVID-19 had an elevated risk of developing new-onset diabetes. The risk was higher in unvaccinated patients than in vaccinated patients.	[35]
2021	Italy	Nearly half of this Italian cohort of COVID-19 patients presented with hyperglycemia. Also, in their study, they reported insulin resistance and new-onset diabetes in recovered COVID-19 patients who did not have a history of diabetes.	[36]
2023	Brazil	Hyperglycemia was higher among COVID-19 patients than non-COVID-19 patients, regardless of diabetes status.	[11]
2024	International	Pediatric patients aged 10 to 19 years old who recovered from COVID-19 were significantly more likely to be diagnosed with new-onset diabetes within 6 months of recovery compared to non-COVID-19 respiratory infection control patients.	[37]
2023	USA	This case study describes a patient admitted to the hospital presenting diabetic ketoacidosis (DKA) one week after receiving the second dose of the Pfizer-BioNTech vaccine. The patient was soon diagnosed with latent autoimmune diabetes (LADA).	[38]

## Data Availability

No new data were created or analyzed in this study. Data sharing is not applicable to this article.
